# Sleep improvements on days with later school starts persist after 1 year in a flexible start system

**DOI:** 10.1038/s41598-022-06209-4

**Published:** 2022-02-18

**Authors:** Anna M. Biller, Carmen Molenda, Giulia Zerbini, Till Roenneberg, Eva C. Winnebeck

**Affiliations:** 1 Institute of Medical Psychology, Ludwig Maximilian University Munich, Munich, Germany; 2 Graduate School of Systemic Neurosciences, Ludwig Maximilian University Munich, Munich, Germany; 3 Department of Medical Psychology and Sociology, University of Augsburg, Augsburg, Germany; 4 Institute of Neurogenomics, Helmholtz Center Munich, Neuherberg, Germany Ingolstädter Landstr. 1, 85764; 5 Present Address: Institute of Psychology, Bundeswehr University Munich, Munich, Germany; 6 Present Address: Institute and Polyclinic for Occupational-, Social- and Environmental Medicine, Ludwig Maximilian University Munich, Munich, Germany; 7 Present Address: Chair of Neurogenetics, Faculty of Medicine, Technical University of Munich, and Institute of Neurogenomics, Helmholtz Center Munich, Munich, Germany

**Keywords:** Neuroscience, Psychology, Neurology

## Abstract

Early school times fundamentally clash with the late sleep of teenagers. This mismatch results in chronic sleep deprivation posing acute and long-term health risks and impairing students' learning. Despite immediate short-term benefits for sleep, the long-term effects of later starts remain unresolved. In a pre-post design over 1 year, we studied a unique flexible school start system, in which 10–12th grade students chose *daily* between an 8:00 or 8:50AM-start. Missed study time (8:00–8:50) was compensated for during gap periods or after classes. Based on 2 waves (6–9 weeks of sleep diary each), we found that students maintained their ~ 1-h-sleep gain on later days, longitudinally (n = 28) and cross-sectionally (n = 79). This gain was independent of chronotype and frequency of later starts but attenuated for boys after 1 year. Students showed persistently better sleep quality and reduced alarm-driven waking and reported psychological benefits (n = 93) like improved motivation, concentration, and study quality on later days. Nonetheless, students chose later starts only infrequently (median 2 days/week), precluding detectable sleep extensions in the flexible system overall. Reasons for not choosing late starts were the need to make up lost study time, preference for extra study time and transport issues. Whether flexible systems constitute an appealing alternative to fixed delays given possible circadian and psychological advantages warrants further investigation.

## Introduction

Teenagers around the world are chronically sleep deprived because their late sleep timing often clashes with early school starts forcing them to get up long before their sleep has come to a natural end. Sleep is timed progressively later during adolescence because teenagers’ internal circadian phase (chronotype) markedly delays^1–3^. At the same time, sleep pressure (the homeostatic load) accumulates more slowly over the day compared to adults or younger children, making teenagers less tired in the evening^4,5^. These biological tendencies are exacerbated by non-biological factors, such as academic pressure or cultural influences to stay up late^6,7^. Evening activities then lead to longer exposure to artificial light at night which increases alertness^8–10^ and further delays circadian rhythms resulting in later sleep timings. Consequently, many students do not get enough sleep during the school week and compensate their sleep loss by oversleeping on weekends. This is often accompanied by a delay of sleep timing on free days—a phenomenon called “social jetlag”^11^. Yet, even with weekend lie-ins, most teenagers do not achieve weekly sleep durations of at least 8 h each night^12,13^, the recommended minimum sleep amount at this age^14^.

The consequences of short sleep are numerous biological and psychological health compromises. In the long-term, chronic sleep deprivation has been linked to metabolic, cardiovascular, and inflammatory diseases^15,16^, to depressed mood and worsened emotional regulation^17–19^, as well as substance use^20,21^. Social jetlag, too, is associated with metabolic syndrome, obesity and depression, as well as increased alcohol consumption and smoking^11,22–25^.

The obvious solution, to simply delay school start times by a fixed amount, has gained much scientific and public attention over the past decades. Positive associations were found for sleep and sleep quality, daytime sleepiness, wellbeing and mood^26,27^, concentration and attention in class, absenteeism and tardiness, and even motor vehicle accidents^28–32^. Nonetheless, policy-uptake is still rare (except for California, USA), also invoking the low evidence level of the findings and unclear long-term benefits as a reason^33,34^. Indeed, the vast majority of studies used a cross-sectional design, which does not allow to track individual changes over time and is prone to cohort effects if not randomized or very carefully adjusted^30,35^. Double-blinding, the gold standard in terms of evidence level, is, of course, inherently unfeasible in this context, and it seems almost impossible to convince schools to participate in randomization^36^. Although there are some real-life settings, such as in Uruguay or Argentina, where students are randomly assigned to morning, middle, and afternoon school shifts^37,38^, this is not the case in most other countries around the world. The few longitudinal studies that exist often covered ≤ 6 months in their follow-ups^30^ (but see^27,39–42^), and are thus prone to seasonal confounding. Furthermore, sleep, mood, and performance have often been assessed via one-off questionnaires, while continuous sleep recordings via daily sleep diaries and especially objective actimetry measures are scarce^27,30,32,43–46^. One notable exception is a recent study by Widome and colleagues who followed students over 2 years and found persisting extended sleep durations (measured with 1 week of actimetry) in students from schools who delayed bell times compared to students in schools which did not change^42^.

We had previously investigated sleep changes and psychological benefits following a switch to a *flexible* start system—a highly overlooked start system that might offer some interesting advantages^47^. Here, we now report on the longer-term effects of this flexible system after 1 year of exposure. The flexible system was established at a German secondary school to provide flexibility on the school start time on a *daily basis*. This means that every single student in 10th–12th grade decides *each day* if they attend the first period at 8AM or if they skip the first period and start at 08:50AM instead. In the rare case of a scheduled free second period, skipping the first period leads to a 10:15AM-start. Non-attended first periods have to be made up for within the same week during free periods or after classes.

Right after the introduction of the flexible system, students had extended their sleep on days with later starts by more than 1 h, reported better sleep quality and slightly less alarm-driven wakings^47^. Nonetheless, compared to baseline, sleep duration had not significantly increased in the flexible system overall. This was caused by students not making full use of the later start option but only choosing to start school later on a median of 2 schooldays per week, and by the fact that there were some infrequent later starts already at baseline. Here, we investigated the situation after 1 year. Did the uptake of the late option increase and thus lead to marked sleep benefits in the flexible system? Did students maintain their large sleep gains on days with later starts? Or did they adjust to the flexible system and delay their sleep times throughout the week?

## Methods and materials

### Study site

The study took place at the Gymnasium Alsdorf (50° 53′ N, 6° 10′ E), a secondary school in a town of ~ 45,000 residents in the West of Germany. A Gymnasium is the most academic of secondary schools in Germany and grants access to higher education after 8–9 years of study and successful completion of the final exam. The school received the German School Award in 2013 for its innovative teaching^48^. It follows an educational system called “Dalton plan” that incorporates daily self-study periods called “Dalton hours” during which students work through a personal 5-week curriculum with a teacher and on a subject of their own choice.

### Change in school start times

The school changed permanently from a fixed start (“conventional system”) to a flexible start (“flexible system”) for older students (grades 10–12) on February 1st, 2016. In the conventional system, the first period started at 8AM. On a median of 1 day/week, depending on students’ individual timetables, classes started with the second period at 8:50AM.

In the flexible system, one of the two daily self-study periods was advanced into the first period (lasting 08:00–08:45AM) and made optional to attend for students in grades 10–12 (for an example timetable see^47^. Students could thus choose daily whether to start at 8AM with the first self-study period or skip it and start at 08:50AM instead (called “9AM” for simplicity). On a median of 1 day/fortnight, students also had a scheduled free second period (08:50–09:50AM), i.e. the chance to turn the 08:50-start into an 10:15-start when skipping the first period (“ > 9AM”). Given the low frequency of 10:15-starts (median 25%, see “Results”), we grouped the two types of later school starts into “ ≥ 9AM-days” and compared those with 8AM-days.

Students had to make up for the skipped first periods throughout the week, using gap periods or adding study time after their last classes (up to the official school closing at 4:15 PM). To be able to start later on all 5 schooldays/week, most students had to make use of both options since their individual schedules did not provide 5 gap periods and 5 early class ends per week.

### Study design

**Figure 1:**
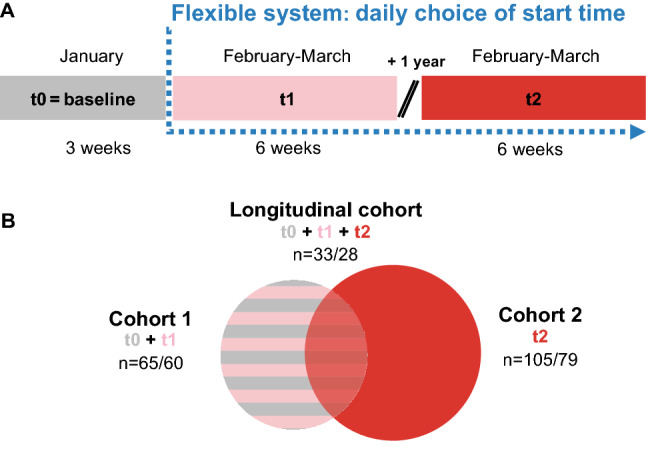
Study design and cohort overview. (**A**) Schematic of longitudinal study design including wave 1, which consisted of 3 weeks of baseline assessment (t0) and 6 weeks in the flexible system (t1), and wave 2 after 1 year again covering 6 weeks of sampling (t2). (**B**) Schematic of the resulting 3 different study cohorts and their respective sample sizes**.** Note that sample sizes vary depending on quality filters applied (see Table 1 and “Participant” section in “Methods” for further information).

Data were collected in two waves that were exactly 1 year apart (Fig. 1A). Wave 1 took place in winter 2016 and consisted of (i) a baseline data collection covering 3 weeks in January (t0, Jan 8th to 31st, 2016) in the conventional system with mainly 8AM-starts, (ii) a data collection for 6 weeks (t1, Feb 1st to Mar 14th, 2016) in the flexible system right after its introduction on Feb 1st, 2016. For the follow-up study (wave 2), we chose the matching photoperiod and time of t1_,_ lasting from Feb 2nd to Mar 20th, 2017 (t2). As the school had remained in the flexible system ever since the introduction, no second baseline just before t2 was carried out. The holiday periods over carnival between February 4th–9th, 2016 and February 23rd–28th 2017 were excluded from the analyses.

### Participants

Written informed consent was obtained from all participants (or their parents/guardians if < 18 years). The study was conducted according to the Declaration of Helsinki and approved by the school board, the parent-teacher association, the school’s student association and the ethics committee of the Medical Faculty of the LMU Munich (#774-16). We used opportunity sampling without specific exclusion criteria. In the first year (t0 and t1), 113 (45%) out of 253 possible students attending 10th–12th grade (14–19 years) signed up, 83 (73%) students provided some data (response rate), of which 65 (70%) passed our minimal quantity and quality filter criteria (cohort 1). In the second year (t2), 162 (71%) out of 227 possible students signed up, 137 (85%) provided data (response rate), of which 105 (77%) passed the minimal filter (cohort 2). Across both years, 33 students passed the minimal filter, hence forming the longitudinal cohort (Table 1). To determine the longitudinal attrition rate, one needs to note that of the 65 students in cohort 1, 16 students graduated after t1 and hence could not participate at t2 (scheduled attrition rate of 34%). Of the 49 students that could have partaken again in t2, 16 provided no or insufficient data at t2 (attrition rate of 33%). Differences in baseline characteristics between the 33 and 16 students were tested and not statistically significant (chronotype, social jetlag, gender, grade level; all p > 0.05), except for age (t(47) = − 2.933, p = 0.005, d = 0.893) with the missing students on average 0.8 years older.

**Table 1:** Composition of study cohorts.

		Cohort 1	Longitudinal cohort		Cohort 2
Time points		t0 and t1	t0 and t1	t2	t2
**Participants**					
Total	n	65	33		105
Females	n (%)	40 (62%)	20 (60%)		73 (70%)
Grade level	n (%) per level 10th/11th/12th	26/23/16 (40/35/25%)	20/13/0 (60/40/0%)	0/20/13 (0/60/40%)	29/38/38 (28/36/36%)
Age	Mean (SD, range)	16.5 (1.2, 14–19)	15.8 (0.9, 14–17)	16.9 (0.9, 15–18)	16.7 (1.1, 15–21)
Chronotype (MSF_sc_; time in h)	Mean (SD, range)	4.6 (0.9, 2.1–7.0)	4.3 (0.7, 2.1–5.9)	4.6 (0.9, 0.8–6.2)	4.7 (1.0, 0.2–8.6)
Social jetlag (h)	Mean (SD, range)	1.8 (0.7, 0.3–3.8)	1.7 (0.6, 0.3–3.1)	1.9 (0.6, 0.5–3.3)	2.0 (0.8, 0.2–6.0)
Sleep duration (h)	Mean (SD, range)	7.6 (0.8, 5.2–8.9)	7.7 (0.8, 5.2–8.8)	7.6 (0.7, 6.1–9.0)	7.7 (0.7, 6.1–9.3)
**Proportion of schooldays with later starts**					
≥ 9AM-use	Median (IQR)	32% (19–55)	39% (20–51)	22% (11–46)	24% (10–47)
**Students reaching ≥ 8 h of sleep in the flexible system**					
8AM-days	%	15%	12%	3%	7%
≥ 9AM-days	%	50%	59%	45%	47%
Schooldays	%	19%	18%	9%	13%
Weekends	%	74%	85%	70%	74%
**Number of students per outcome**					
Sleep	8AM vs. ≥ 9AM-days	n = 60	n = 28		n = 79
	Conv. vs. flexible system	n = 65	n = 33		n = 105
Psychological benefits					n = 91–93

Displayed are cohort characteristics after standard filter criteria. An additional filter (see “Participant” section in “Methods”) was applied for comparisons between 8AM and ≥ 9AM-days, which reduced cohort 1 to 60 students, the longitudinal to 28 students, and cohort 2 to 79 students.

n, number of individuals; SD, standard deviation; IQR, interquartile range; conv., conventional.

Minimal filter criteria were: (i) sleep information for ≥ 5 schooldays and ≥ 3 weekend days at each time point and (ii) congruent, plausible data (more detailed information in^47^). For 8AM or ≥ 9AM-start comparisons, we additionally filtered for at least 2 8AM-days and at least 2 ≥ 9AM-days per person. After this additional filter, a total of 60 participants remained in cohort 1, 79 in cohort 2, and 28 in the longitudinal cohort. All students from the longitudinal cohort were granted promotion to the next grade level from wave 1 to wave 2.

### Outcome measures

#### Sleep diary

We used a daily sleep diary (provided online via LimeSurvey.org) based on the μMCTQ^49^ (a short version of the Munich Chronotype Questionnaire) and adapted it to a German student population by changing the formal you (“Sie”) to the informal you (“Du”) and work days to schooldays. Students provided sleep onset (note: not bedtime) and offset (wake time) of their past night’s sleep, whether they were woken by their alarm clock (yes/no), the type of day they woke up (schoolday or free day), when they started school (8AM, 9AM or > 9AM), and their subjective sleep quality (rated on a 10-point-Likert scale from 1 = “very bad” to 10 = “very good”). The questionnaire did not cover any naps during the day. Although daily population of the online sleep diary was encouraged, students could also fill in data in retrospect if they had missed a day or more (they reported to have kept an offline log from which they copied their sleep timings). For more details see^47^.

#### Survey

We developed a 17-item paper–pencil survey about the flexible system, which was distributed at the end of t2 and filled out by ~ 90% of cohort 2. Because some students did not answer all questions on the survey, the sample size ranged from 91 to 93 depending on the item. The first 7 items of the survey asked whether (i) students were satisfied with the flexible system (yes/no), (ii) they would rather have the old system with fixed school starts back (yes/no), (iii) it was difficult for them to go to school at 8AM (never/most of the time/always), (iv) it was easier to go to school at 9AM compared to 8AM (never/most of the time/always), (v) how often (0 days/1–2 days/3–4 days/5 days) and (vi) on which days of the week they attended the first period at 8AM (Mo/Tu/We/Th/Fr), and (vii) reasons for starting school at 8AM. Answer options for (vii) were to mark at least one of nine alternatives (easier to study/easier to get to school/additional study time/friends/specific teacher/specific subject/fulfill self-study quota/parents/late school end) and/or to name other reasons.

The last 10 items asked for ratings on 8AM versus ≥ 9AM-days. Questions were about (i) sleep duration (h), (ii) sleep quality (1 = "bad", 5 = "good"), (iii) number of schooldays with alarm-driven waking (0–5 days), (iv) how tired the students felt (1 = "not at all", 5 = "very"), (v) ability to concentrate in class (1 = "bad", 5 = "good"), (vi) ability to study at home after school (1 = "bad", 5 = "good"), (vii) motivation to actively take part in class (1 = "not at all", 5 = "very"), (viii) how well they remembered new class content (1 = "not at all", 5 = "very"), and (ix) attitude towards school (1 = "negative", 5 = "positive"). Items (ii) and (iv)–(ix) were scored on a 5-point Likert scale.

### Data analysis

Analyses were performed in SPSS Statistics (IBM, versions 24 and 25), R (versions 3.6.1 and 3.6.3) and R studio (versions 1.1.463, 1.2.1335 and 1.2.5042). Graphs were produced using Graph Pad Prism (versions 6 and 7) and the R package *ggplot2*^50^. Main figures (except Fig. 3) show results from the longitudinal cohort (n = 28–33); results from cohort 2 (n = 79–105) are provided in the text and SI.

#### Sleep data

Daily sleep data from diaries were aggregated as mean per person for 10 conditions: at t0 for schooldays and weekends; and at t1 and t2 for schooldays, weekends, 8AM-days, and ≥ 9AM-days. From these aggregates, we derived the following variables as per equations below for each of the time points (t0, t1, t2): average daily sleep duration during the week (SD_week_), chronotype as midsleep on free days (MSF) corrected for oversleep (MSF_sc_), and social jetlag (SJL); for t1 and t2 only: absolute difference between ≥ 9AM-days and 8AM-days for variables of interest (DELTA x), frequency of alarm-driven waking, and frequency of ≥ 9AM-starts.

$${\mathrm{SD}}_{{\mathrm{week}}}={(\mathrm{SD}}_{{\mathrm{schooldays}}}*5+ {\mathrm{SD}}_{{\mathrm{free}} \, \mathrm{days}}*2)/7$$

$$\mathrm{MSW}= {\mathrm{SleepOnset}}_{{\mathrm{schooldays}}}+{\frac{1}{2}\mathrm{SD}}_{{\mathrm{schooldays}}}$$

$$\mathrm{MSF}= {\mathrm{SleepOnset}}_{{\mathrm{free}} \, \mathrm{days}}+{\frac{1}{2}\mathrm{SD}}_{{{\mathrm{free}}} \; \mathrm{{days}}}$$

$${\mathrm{MSF}}_{sc}= {\mathrm{SleepOnset}}_{{\mathrm{free}} \, \mathrm{days}}+{\frac{1}{2}\mathrm{SD}}_{{\mathrm{week}}}$$

$$\mathrm{SJL}=\mathrm{MSF}-\mathrm{MSW}$$

$$\text{DELTAx}={\text{x}}_{9{\text{AM-days}}}-{\text{x}}_{8{\text{AM-days}}}$$

$$\text{Frequency} \; \text{of} \; \text{alarm-driven} \; \text{waking} = ({\text{n}}_{{\text{alarm-driven} \; \text{waking}}_{\text{flex}}}/{\text{n}}_{{\text{schoolday-entries}}_{\text{flex}}})*100$$

$$\text{Frequency} \; \text{of} \ge 9\text{AM-starts}= ({\text{n}}_{{9\text{AM-starts}}_{\text{flex}}}/{\text{n}}_{{\text{schoolday-entries}}_{\text{flex}}})*100$$

#### Statistical analyses

Unless indicated otherwise, descriptive statistics are reported as mean ± standard deviation and test statistics, p-values and effect size measures are abbreviated as follows: *t*, t-test; *Z*, Wilcoxon signed-rank test; *F*, ANOVA; *r*, Pearson correlation; *rho*, Spearman rank correlation; *p*, significance level; *d*, Cohen’s d; *d*_*z*_*,* Cohen’s d for paired t-tests; *b*, unstandardized coefficient; *beta*, standardised coefficient. Significance levels were set to *p* < 0.05 for all statistical analyses. All data were tested on normality (histograms, QQ plots, Shapiro–Wilk’s test) and sphericity (Mauchley’s test; Greenhouse–Geisser corrections used for ANOVA if violated). If normality was violated, non-parametric tests were performed except for ANOVA analysis since violations were marginal.

Group difference for attrition groups were tested via independent t-test (chronotype, social jetlag, age) or Chi squared test (gender, class).

For sleep variables in the longitudinal cohort, we performed 1-way repeated measures ANOVAs with the factor time point (t0/t1/t2), 2-way repeated measures ANOVAs with the factors time point (t1/t2) and school start (8AM/ ≥ 9AM-days), and with the factors time point (t0/t1/t2) and day (schooldays/weekend). For sleep variables in cohort 2, paired t-tests (two-sided) were run for school start (8AM/ ≥ 9AM-days) and days (schooldays/weekend), and Wilcoxon signed rank test for sleep quality and survey items. Gender differences in sleep variables were assessed via 2-way mixed ANOVA with gender (female/male) and time point (t1/t2), and via linear regression (including the covariates grade level, chronotype and frequency of ≥ 9AM-starts) for DELTA sleep duration/onset/offset using the *nlme* package in R^51^. ANOVA results are presented above each graph (main effects and interaction). If the main interaction was significant, we interpreted (and thus provide) only the simple effects instead of the main effects. In cases of three levels within one factor, necessary post hoc tests were carried out using Bonferroni corrections.

Pearson and Spearman rank correlations were performed to assess associations between DELTA sleep duration and chronotype or frequency of ≥ 9AM-starts, respectively. Frequency of alarm driven waking was analysed using logistic regression (*lme4* package R^52^). Due to a large ceiling effect, we dichotomised this variable based on a median split at 100%-use (< 100%: “less use”) and accommodated the repeated measures nature of the data by including ID as a random effect in a mixed regression model. Gender was included as covariate (males were woken more often by an alarm than females in the flexible system) but gender did not reach statistical significance.

## Results

During the first wave of our study^47^, we had monitored students’ sleep in detail via diaries and actimetry for 3 weeks during baseline (= t0) and 6 weeks immediately after the change into the flexible system (= t1). To investigate the longer-term effects, we conducted the second wave after exactly 1 year (t2) at the same photoperiod as t1 to optimally control for seasonal effects. After 6 weeks of daily sleep diary, we also surveyed subjective wellbeing and psychological functioning on days with early versus later starts.

We allowed students to take part in wave 2 (Fig. 1A) irrespective of their participation beforehand, so our study eventually consisted of three cohorts (Fig. 1B and Table 1): (i) cohort 1 provided sleep data at t0 and t1 (n = 60–65), (ii) cohort 2 provided sleep and survey data only at t2 (n = 79–105), and (iii) the longitudinal cohort provided sleep data throughout from t0-t2 (n = 28–33). The samples sizes within each cohort varied due to different filters employed for different analysis questions (see “Participant” section in “Methods”).

### Frequency of later starts (≥ 9AM-use)

Notably, our participants accumulated fewer late starts per week than expected. We had observed this for cohort 1^47^ but now saw this confirmed in cohort 2, where participants (n = 105) chose to skip the first period only on a median of 24% of their schooldays (IQR: 10–47), which equates to 1.2 ≥ 9AM-days per 5-day school week. Similarly, the longitudinal cohort (n = 33) had a median frequency of late starts (“ ≥ 9AM-use”) of 39% (20–51) and 22% (11–46) during t1 and t2, with no systematic difference between the time points (Z = − 1.653, p = 0.098). Importantly, ≥ 9AM-use varied drastically between individual participants from 0 to 100% of their schooldays, with 8:50AM-starts making up the majority of later starts per person and 10:15AM-starts, due to a second free period, only 25% (median, IQR: 6.3–60).

### Sleep on days with later school starts

In the following, we present analyses *within* the flexible system comparing days with early school starts (“8AM-days”) to those with later starts (“ ≥ 9AM-days”).

#### Student slept longer and better on days with later school starts—an improvement persisting over 1 year

**Figure 2:**
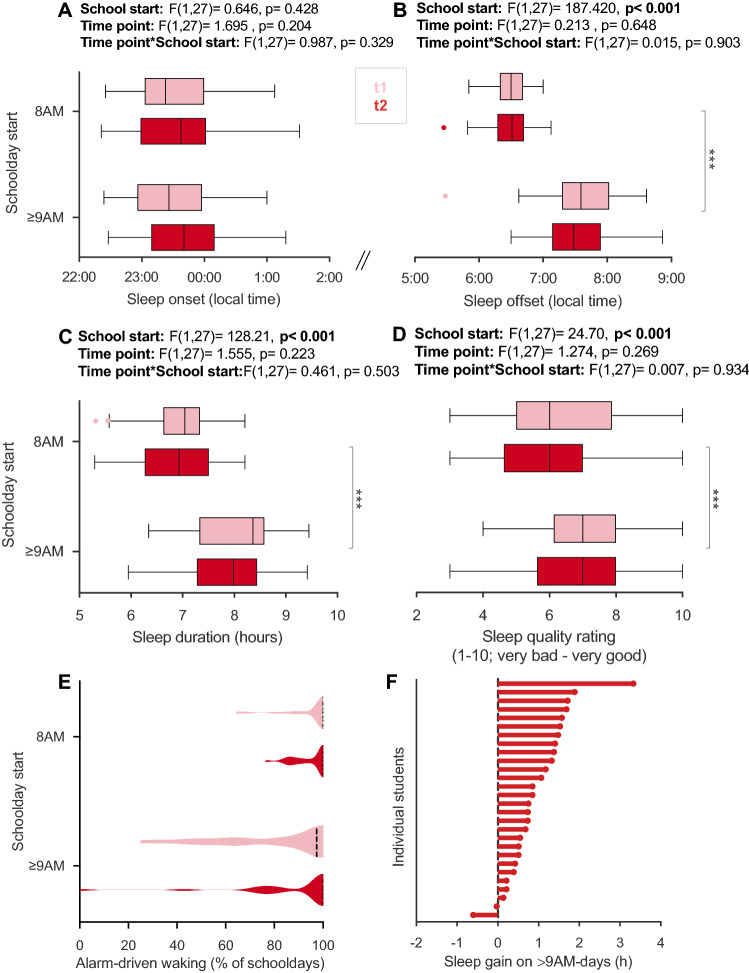
Comparison of sleep parameters between 8AM-days and ≥ 9AM-days in the flexible system. (**A**–**F**) Sleep parameters from the longitudinal cohort (n = 28) comparing 8AM and ≥ 9AM-days at t1 (light red) and t2 (dark red) intra-individually. (**A**) Average sleep onset, (**B**) offset, (**C**) duration, and (**D**) quality on 8AM versus ≥ 9AM-days in the flexible system across time points. Results of two-way repeated measures ANOVA with the within-subject factors school start (8AM/ ≥ 9AM) and time point (t1/t2) are reported above each graph. Brackets indicate statistically significant post-hoc comparisons. (**E**) Proportion of schooldays with alarm-driven waking. (**F**) Sleep gain on ≥ 9AM-days at t2 for each student. Depicted is the absolute difference in average sleep duration between 8AM and ≥ 9AM-days. Positive values mean longer sleep on ≥ 9AM-days. Dashed lines in violin plots show medians. All boxplots are Tukey boxplots. *p < 0.05, **p < 0.01, ***p < 0.001.

How was students’ sleep altered by later school start times in the flexible system over 1 year? We showed previously that, right after the introduction of the flexible system, students from cohort 1 slept about one hour longer on ≥ 9AM-days by maintaining their sleep onset but delaying their sleep offset^47^. After 1 year, we found the same sleep gain of ~ 1 h for cohort 2 and, importantly, also in the longitudinal cohort across both time points. Repeated measures ANOVAs in the longitudinal cohort (n = 28) showed that sleep onsets did not differ with school start time or time point (Fig. 2A), whereas sleep offsets were on average 61 min (± 47) later (Fig. 2B), and students hence slept 62 min (± 47) longer on ≥ 9AM-days compared to 8AM-days across both time points (Fig. 2C,F, full statistics in Figures). Findings from cohort 2 (n = 79) tally with this pattern: sleep onsets on 8AM and ≥ 9AM-days were comparable (t[78] = − 1.87, p = 0.065; d_z_ = 0.210), while wake up times were significantly later on ≥ 9AM-days (t[78] = − 19.75, p < 0.001, d_z_ = 2.222), which resulted in 60 min longer sleep durations on those days (t[78] = − 10.83, p < 0.001, d_z_ = − 1.218). This large sleep gain likely results from ≥ 9AM-days incorporating not only 8:50-starts (75%) but also some 10:15-starts (25%).

Furthermore, subjective sleep quality was improved on ≥ 9AM-days by 1 point on a 10-point Likert scale for cohort 1^47^ and cohort 2 (n = 79, Z = − 5.874, p < 0.001, d = − 1.761), and also longitudinally across time points (n = 28, Fig. 2D). In addition, the extensive use of alarm clocks remained slightly reduced on ≥ 9AM-days also 1 year into the system (Fig. 2E). Just as in cohort 1^47^, the odds for less alarm-driven waking were increased in cohort 2 (n = 79, OR = 1.9, 95% CI = 1.3–4.1) and showed a similar qualitative pattern also in the longitudinal cohort (n = 28; Fig. 2E), demonstrating that a natural waking was more likely when school started later.

#### Students reported profound improvements in cognitive and psychological parameters on days with later school starts

**Figure 3:**
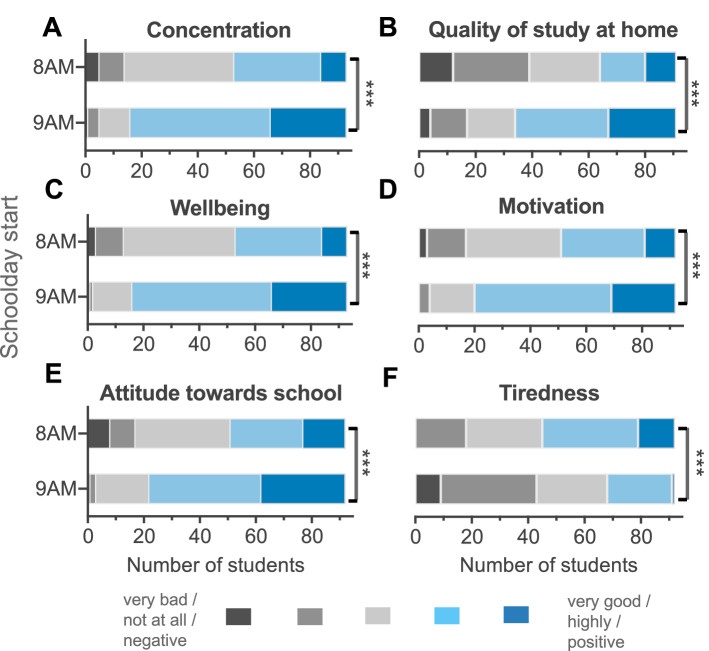
Comparison of subjective psychological benefits between 8AM-days and ≥ 9AM-days in the flexible system. Results from the survey at end of wave 2 asking cohort 2 for the following ratings: (**A**) ability to concentrate during class (Z = 6.419, d = 1.784, n = 93), (**B**) quality of study at home after school (Z = 6.055, d = 1.643, n = 91), (**C**) general wellbeing (Z = 6.559, d = 1.855, n = 93), (**D**) motivation to attend school (Z = 5.927, d = 1.572, n = 92), (**E**) attitude towards school (Z = 5.896, d = 1.545, n = 92), and (**F**) tiredness during class (Z = 5.419,d = 1.369, n = 92). Wilcoxon signed rank test, *p < 0.05, **p < 0.01, ***p < 0.001.

To assess psychological benefits, we used survey data from the end of t2, which were provided by 90% of cohort 2. Students’ subjective ratings of their sleep, cognition and well-being on 8AM-days compared to ≥ 9AM-days showed statistically significant improvements in all areas assessed (n = 91–93; full statistics in Fig. 3). On days with later starts, students felt generally better, less tired during class, more motivated to actively take part in class, and were better able to concentrate. Students also reported a more positive attitude towards attending school and higher quality of self-study after school.

#### Girls maintained their sleep benefit from later school starts more than boys after 1 year in the flexible system

We wondered whether specific students benefitted more or less than others from later starts. Therefore, we assessed the relationship of chronotype, ≥ 9AM-use and gender with the core sleep benefit, the sleep gain on ≥ 9AM-days (the difference in sleep duration between ≥ 9AM and 8AM-days).

**Figure 4:**
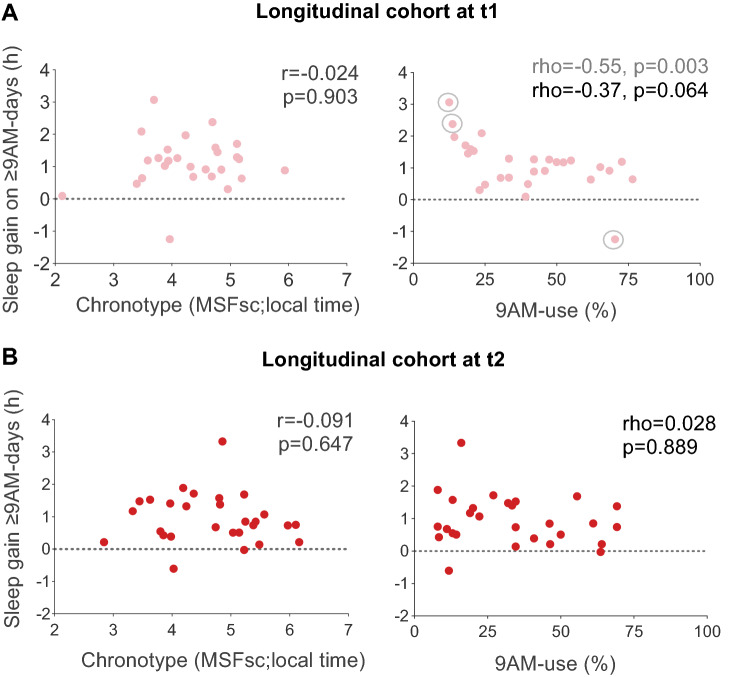
Inter-individual differences in sleep gain on ≥ 9AM-days. Shown are relationships between chronotype (MSF_sc_; local time) or frequency of ≥ 9AM-starts (% of schooldays with later starts) with sleep gain on ≥ 9AM-days. Sleep gain was quantified as the absolute difference in sleep duration between ≥ 9AM and 8AM-days, with positive numbers indicating longer sleep duration on ≥ 9AM-days. Data are from the longitudinal cohort (n = 28) during (**A**), t1 (light red) and (**B**), t2 (red). Results of Pearson (r) and Spearman (rho) correlations are indicated. Tukey outliers in sleep gain, which over-proportionally influence the correlation with 9AM-use at t1 (right panel in **A**), are marked with grey empty circles, and correlation results including (grey) and excluding outliers (black) are provided.

In the longitudinal cohort (n = 28; see further below for cohort 2), 93% of students experienced a sleep gain on ≥ 9AM-days across both time points (Fig. 2F), so the sleep benefit was close to universal. Chronotype was not correlated with sleep gain (t1: r = − 0.024, p = 0.903; t2: r = − 0.091, p = 0.647), i.e. both early and late chronotypes appear to have benefitted equally from later starts (Fig. 4A,B). We had already observed this in cohort 1^47^ and interpreted it as the consequence of the severe sleep deprivation in adolescent students which afflicts even earlier chronotypes. Similarly, no matter how often the students attended school later, their sleep gain on ≥ 9AM-days did not seem systematically affected. Although correlations indicated smaller gains with more frequent ≥ 9AM-use at t1 (rho = − 0.55, p = 0.003), this was mainly driven by two over-benefitting individuals with low ≥ 9AM-use and one under-benefitting individual with high use—all three identified as outliers already in our wave-1-analyses^47^. Without these three Tukey outliers, the relationship was smaller and statistically non-significant (rho = − 0.37, p = 0.064). There was also no correlation between sleep gain and ≥ 9AM-use during t2 (rho = 0.028, p = 0.889; Fig. 4A,B).

**Figure 5:**
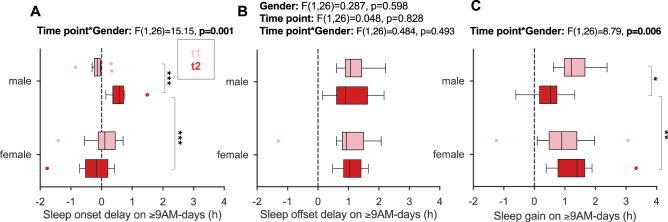
Gender differences in sleep onset, offset and duration on ≥ 9AM-days versus 8AM-days in the flexible system. Depicted is the absolute difference between 8AM and ≥ 9AM-days in (**A**)**,** average sleep onset (sleep onset delay) and (**B**)**,** average sleep offset (sleep offset delay) and (**C**)**,** average sleep duration (sleep gain) per individual from the longitudinal cohort (n = 28), with positive numbers indicating higher values on ≥ 9AM-days. Results of two-way mixed ANOVAs with the between-subjects factor gender (female/male) and the within-subjects factor time point (light red = t1/red = t2) are reported above each graph. Given the significant interaction effect on sleep onset delay, main effects are not reported, instead statistically significant post-hoc comparisons are indicated. See main text and Supplementary Tab. S1 for detailed effect sizes. All boxplots are Tukey boxplots. *p < 0.05; **p < 0.01; ***p < 0.001.

In contrast, gender showed a clear association with sleep gain after 1 year: both genders enjoyed similar sleep gains during t1, as also found in cohort 1^47^, but boys clearly reduced their sleep gain during t2 from 1.3 h (± 0.53) to 0.5 h (± 0.53, detailed statistics in Fig. 5C and Supplementary Tab. S1). Follow-up analyses revealed that the reduced sleep gain in boys resulted from a delay in their sleep onsets on ≥ 9AM-days compared to 8AM-days (Fig. 5A), while their offset times were unaltered during t2 (n = 28; Fig. 5B, statistics in Fig. 5 and Supplementary Tab. S1).

The bigger sample size of cohort 2 (n = 79) allowed us to address all the above relationships together in single regression models, in particular the reasons for the gender disparity. Besides gender, chronotype and ≥ 9AM-use, we also included grade level (inherently incorporating age) as predictors for sleep gain, sleep onset delay and sleep offset delay (the differences between ≥ 9AM and 8AM-days; Supplementary Tab. S2). The regression results corroborated all observations from the longitudinal cohort showing that only gender had a significant association with any of the outcomes, namely sleep gain and sleep onset delay (Supplementary Tab. S2). Boys reduced their sleep gain on average by 0.52 h (b = − 0.52, p = 0.010, r =  > 0.6), which was driven by a delay in their onset on ≥ 9AM-days by 0.53 h (b = 0.53, p < 0.001, r > 0.6), while their offset was unchanged (b = 0.01, p = 0.942, r = 0.07). Sensitivity analyses indicated that this effect was not just driven by the longitudinal cohort comprising 35% of cohort 2. Taken together, while most inter-individual differences did not systematically associate with sleep gains, boys showed a delay in sleep onset and thus displayed a smaller sleep gain on ≥ 9AM-days after 1 year in the flexible system.

### Sleep in the flexible system versus baseline

Despite obvious improvements in sleep and subjective parameters on ≥ 9AM-days also after 1 year, it is essential to determine if these also translated into better sleep in the flexible system overall. Based on our analyses of cohort 1^47^, this was largely not the case during the first 6 weeks after the introduction of the flexible system. Most likely, the limited ≥ 9AM-use in combination with occasional late starts during baseline reduced improvements by the flexible system compared to the conventional system. But did long-term effects emerge after 1 year of exposure in the flexible system?

#### Students did not extend their sleep in the flexible system overall

**Figure 6:**
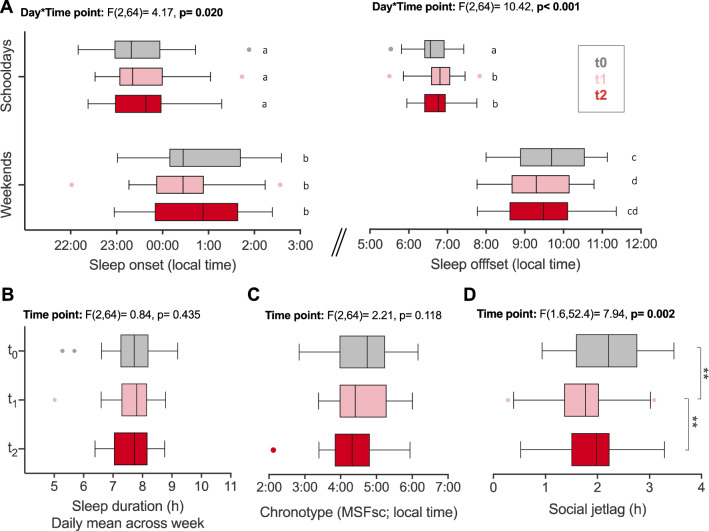
Comparison of sleep parameters across school start systems. Sleep parameters from the longitudinal cohort (n = 33) comparing the conventional start system at baseline (t0, grey) with the flexible system during t1 (light red) and t2 (dark red). (**A**) Average sleep onset and offset on schooldays and weekends. Results of two-way repeated measures ANOVAs with the factors day (schooldays/weekends) and time point (t0/t1/t2) are provided. Given the significant interaction effect, main effects are not reported. Letters indicate results of post-hoc tests on simple contrasts, with data marked by different letters demonstrating significant differences. (**B**) Average daily sleep duration across the week (weighted for 5 schooldays and 2 weekend days), (**C**) average chronotype, (**D**) average social jetlag. Results of one-way repeated measures ANOVAs across time points are presented above each graph. Brackets indicate statistically significant post-hoc comparisons. All boxplots are Tukey boxplots. See main text and Supplementary Tab. S3 for detailed effect sizes. *p < 0.05, **p < 0.01, ***p < 0.001.

Analyses in the longitudinal cohort (n = 33) revealed that students’ sleep was not improved compared to baseline even after 1 year in the flexible system. Despite small delays in sleep offset on schooldays (Fig. 6A, detailed statistics in Fig. 6 and Supplementary Tab. S3), sleep duration on schooldays and across the week were not significantly increased at t1 or t2 compared to t0 (Fig. 6B). Students still only slept 7.6 h (± 0.65) on a daily average across the week (including weekend catch-up sleep) at t2, a sleep duration below the recommended 8–10 h for this age group^53^. Students’ chronotype remained expectedly late across all time points (Fig. 6C), and there was still a substantial difference between sleep timing on schooldays and weekends (Fig. 6A; Supplementary Tab. S4 for similar results in cohort 2). Students’ social jetlag, which quantifies this typical shifting between the ‘schoolday-time zone’ and the ‘weekend-time zone’, although reduced at t1 by 30 min (± 0.62, p = 0.002), was indistinguishable from baseline after 1 year (p = 0.256; Fig. 6D). So, the mild reduction in social jetlag experienced immediately after entering the system was lost later on, emphasizing that there was no widespread improvement in sleep under the low ≥ 9AM-use in the flexible system.

## Discussion

Teenagers show restricted sleep on schooldays and catch-up-sleep on weekends. Early school starts are a major determinant of this pattern, thereby impacting students’ daily lives and their future trajectories. Most studies that looked at delayed school starts and sleep improvements were cross-sectional (and thus could not track individual differences over time) and analysed outright and fixed delays in start times. Here, we investigated whether a flexible school start system allowed teenagers to reduce their sleep deprivation long-term, and whether this system was also associated with subjective improvements in psychological parameters.

The few studies that recorded sleep changes longitudinally after a delay in school start times reported mixed results. Bowers and Moyer determined in a meta-analysis^30^ that all five longitudinal studies examined showed sleep extensions after a school start delay, and this benefit persisted until the follow-up period at 0.25 to 6 months after the delay^31,43,46,54,55^. Lo et al. also tracked sleep after a 45-min delay and found a delay in bedtime of 23 min which was sustained after 9 months^27^. Meltzer et al. followed middle and high school students around a delay of 40–70 min via anonymous surveys and found increased sleep durations of 29–45 min after 6 months, which were only slightly reduced (3–9 min) after 18 months due to slightly later sleep onsets especially in older students^56^. In contrast, Thacher and Onyper showed that a 20-min sleep extension after a 45-min delay disappeared after 1 year because students delayed their sleep times^40^. Das-Friebel et al. also provided evidence that students merely shifted their sleep timing to later and thus did not benefit from their 20-min school delay after 1 year^41^.

Here, in the flexible start system compared to the conventional start system, we found no overall shift in sleep timing but also no net sleep gains, which is probably connected to the low uptake of later starts of 1–2 days per week on average (although individual uptake ranged from 0 to 100%) and to occasional later starts already occurring during the conventional system. We had identified three main reasons for this low uptake via survey answers during wave 1: students could not fulfil their quota of 10 self-study periods per week without otherwise getting home later in the afternoon (75%), it was easier to get to school for the 8AM-start (40%), and students wanted to have more time to study (27%)^47^. During wave 2, these reasons remained the most common ones (54%, 37%, 50% respectively) for going early—although yet another year later the uptake of the late-start option apparently rose to a median of 79% (IQR = 70–86), i.e. 4 days per week, according to data provided by the school. It is therefore likely that the temperate use of the flexible starts during our recording period underlies the persistent absence of sleep benefits in the flexible system in our sample. Thus, more late starts are probably required to translate into net sleep benefits in a flexible system. Alternatively—or in addition—the flexible system might have compensated a potential delay in sleep with increasing age or adolescence^57,58^ and the absence of a net change in sleep between all time points is actually a success as it prevented a worsening. Longitudinal observational data without a control group, however, are unfortunately not suited to answer this question.

When not comparing *between* systems but *within* the flexible system, our results demonstrate that sleep duration on ≥ 9AM-days remained increased on average by 1 h even after 1 year, and that ≥ 9AM-starts were subjectively helpful for students across many psychological domains. The sleep and psychological effects might be either downstream of each other (e.g. longer and better sleep improving well-being and concentration or vice versa) or parallel improvements (e.g. more self-determination in the flexible system improving both sleep and psychological aspects in day-time functioning). The finding that almost every single student profited from a later start highlights the pervasiveness and severity of sleep deprivation in this age group.

Importantly, while girls’ sleep benefit on ≥ 9AM-days was completely sustained over the follow-up period, boys’ sleep gain was reduced after 1 year since they fell asleep later on ≥ 9AM days than on 8AM-days at t2. This could be a cohort effect of the small longitudinal cohort, but the larger cohort 2, which had a similar gender ratio, showed the same pattern. The delay in sleep onsets for boys but not for girls is a central finding, since avoiding delays in sleep onsets is key to long-term success of later school start times, both flexible and fixed. Our analyses revealed no effects of chronotype or frequency of later starts on this delay. We can thus only speculate about the possible biological, psychological and behavioural reasons explaining the observed gender difference, ranging from different circadian light sensitivities^59^ to (un)consciously differing sleep hygiene or pre-bed activities (e.g. bed procrastination that has been shown to be higher in male students^60^). It is clear that this gender difference after 1 year raises many central questions and might underlie the contradictory findings from the few previous longitudinal studies (with e.g. all-girls samples^27^ or few gender analyses), highlighting the urgent need for long-term follow-ups of sleep timing adjustments with differential effects.

Despite the reduction in boys’ sleep gain on ≥ 9AM-days, the benefits of later school starts within the flexible system were generally immense: while only 3% to 15% of students across all cohorts reached the minimal amount of 8 h required for healthy sleep in teenagers^53^ on 8AM-days, 45–59% of students enjoyed at least 8 h of sleep on ≥ 9AM-days (Table 1). Although students still did not get the recommended 8-10 h on schooldays overall, this demonstrates that later starts constitute a move in the right direction. Sleep durations on ≥ 9AM-days got closer to more optimal levels, which we otherwise only observed on weekends when 70–85% of students in our sample reached at least 8 h. Other studies with fixed delays found similar effects only when school started much later, such as in the afternoon^61,62^.

What about other important aspects of sleep—beyond duration—in the flexible system? Subjective sleep quality and the amount of alarm-free waking were both increased on later days. The question around sleep regularity/variability, however, is less easily answered. In line with the relatively tightly regulated schedule during the schoolweek, the biggest variation in sleep timing and duration in our sample occurred between schooldays and weekends. Social jetlag quantifies this variability based on the differences in midsleep times between free days and schooldays which is also influenced by differences in sleep duration^63^. During wave 1, social jetlag was only slightly reduced in the flexible system compared to baseline^47^ and this did not persist until wave 2—presumably because students did not take the late-start option often enough. If taken more often, later starts could substantially increase schoolweek sleep duration to the extent that oversleeping on weekends might be avoided thus reducing social jetlag, i.e. variability in sleep timing and duration. It could be argued, though, that higher uptake would increase sleep variability *within* the schoolweek. However, since the magnitude of the introduced variability of ~ 1 h in duration is small (far less than that observed in college students^64^) and predominantly only affects sleep offset timing, one could imagine that together with the potential reduction of the schoolweek-weekend difference, the flexible system might thus actually lead to a net reduction in sleep variability overall when used sensibly. Future studies should investigate this unique and interesting aspect in more detail.

An obvious bonus of the flexible system was that students themselves also liked it. They were more motivated to go to school, they rated their concentration and motivation higher during class, and generally felt better on ≥ 9AM-days. These are also prerequisites for good academic learning and achievement. Nonetheless, neither the flexible system nor the frequency of 9AM-use or sleep gains were significantly associated with an improvement in students’ grades, as analysed in detail in an accompanying manuscript^65^.

Our study has some limitations that have not yet been mentioned. Sleep analyses were solely based on subjective diaries entries. However, importantly, diary data corresponded very well to objective activity data in cohort 1 (r = 0.8–0.9)^47^, and other studies report similar correlations^66,67^, so we assume faithful reporting from our sample. Furthermore, our sleep calculations did not consider potential naps and hence might underestimate the total sleep duration in some students. Finally, we also did not have data on the socioeconomic background of our participants. However, students attending Gymnasium (the most academic type of school in Germany) tend to be from families with higher socio-economic status, and often at least one parent has a similar educational level (65.9% of parents obtained A-levels, and 22.2% a General Certificate of Secondary Education equivalent^68^).

In conclusion, students in our sample showed clear subjective psychological benefits in several domains and were able to longitudinally maintain a 1-h sleep gain but only on days with later starts. No overall improvement in sleep duration was observed in the flexible system but flexible start times could still become an interesting alternative to later starts when further improved (e.g. more late starts, better timetabling) given its potential advantages over fixed delays. Future studies should include bigger sample sizes and control groups and need to investigate cognition, learning and motivation in more detail. This also includes increasing our understanding whether teaching students to take responsibility, which incorporates to decide for themselves when to learn and to some extent when to start school, indeed increases their motivation, investment, and wellbeing, and can thus have potential indirect effects on their sleep quality and learning.

### Preprint

A previous version of this manuscript was published as a preprint^69^.

## Supplementary Information

Supplementary Tables.

## Data Availability

Data were collected with a consent form that prohibits online deposition of data for open access sharing. This prohibition was implemented to protect participants’ privacy in a cohort where most individuals are well-acquainted with each other, and peers or teachers might identify participants. Data are available from the corresponding author upon reasonable request.
