# Supplementary material for: Sleep improvements on days with later school starts persist after 1 year in a flexible start system
Source: Sci Rep. 2022 Feb 18;12:2787. doi: 10.1038/s41598-022-06209-4 (PMC8857191; doi:10.1038/s41598-022-06209-4)
Supplement: Supplementary file 1 — Supplementary Tables. [file 41598_2022_6209_MOESM1_ESM.pdf]

Supplementary information for

Sleep improvements on days with later school starts persist after one year in a flexible system

Authors:

Anna M. Biller[1,2+#], Carmen Molenda[1#], Giulia Zerbini[1,3], Till Roenneberg[1§] & Eva C. Winnebeck[1$*]

[#]equal contribution
*corresponding author

Affiliations:

[1] Institute of Medical Psychology, Ludwig Maximilian University Munich, Munich, Germany
[2] Graduate School of Systemic Neurosciences, LMU Munich, Germany
[3] Department of Medical Psychology and Sociology, University of Augsburg, Augsburg, Germany

+ current address: Institute of Psychology, Bundeswehr University Munich, Germany

§ current address: Institute and Polyclinic for Occupational-, Social- and Environmental Medicine, Ludwig Maximilian University Munich, Munich Germany

$ current address: Chair of Neurogenetics School of Medicine, Technical University of Munich, and Institute of Neurogenomics, Helmholtz Center Munich, Munich, Germany

Correspondence should be addressed to:

Eva Winnebeck, eva.winnebeck@helmholtz-muenchen.de, Institute of Neurogenomics, Ingolstädter Landstr. 1, 85764 Neuherberg, Germany

This PDF file includes:

Tables S1-S4

Supplementary Table S1. **Sleep differences between time points and gender in the longitudinal cohort (post-hoc comparisons relating to Fig. 5).** Two-way mixed ANOVAs were run for sleep onset delay, sleep offset delay, and sleep gain on ≥9AM-days respectively with the within-factor time point (t1/t2) and between-factor gender (male/female). In case of significant interaction of both factors, simple effects were indicated instead of interpreting the main effects. Data presented are mean ± standard deviation from the longitudinal cohort (n=28). η^2, (partial) eta squared; d, Cohen's d.

Sleep onset delay on ≥9AM-days

	**t1**	**t2**	Main effect:<br>*Gender*	Simple effects:
**Male**	-0.13 ± 0.30	0.6 ± 0.34	-	F(1,26)=14.030<br>**p=0.001,**<br>**η²=0.350, d= 1.468**
**Female**	0.052 ± 0.51	-0.22 ± 0.54		F(1,26)=2.629<br>p=0.117<br>η²=0.092, d=0.637
Main effect:<br>*Time point*	-		Main interaction<br>*Time point*Gender*:<br>F(1;26)=15.155<br>**p=0.001**<br>**η²=0.368, d=1.526**	
Simple effects:	F(1,26)=1.195<br>p=0.284<br>η²=0.044, d=0.127	F(1,26)=20.888<br>**p=0.000**<br>**η²=0.445, d=1.791**		

Sleep offset delay on ≥9AM-days

	**t1**	**t2**	Main effect:<br>*Gender*	Simple effects:
**Male**	1.20 ± 0.49	1.06 ± 0.62	F(1,26)=0.287<br>p= 0.596, η²=0.011<br>d=0.211	-
**Female**	1.01 ± 0.76	1.08 ± 0.35		-
Main effect:<br>*Time point*	F(1,26)= 0.048<br>p=0.828<br>η²=0.002, d=0.090		Main interaction<br>*Gender*Time point*:<br>F(1,26)=0.484<br>p= 0.493,<br>η²=0.253,d=1.164	
Simple effects:	-	-		

Sleep gain on ≥9AM-days

	**t1**	**t2**	Main effect:<br>*Gender*	Simple effects:
**Male**	1.33 ± 0.53	0.47 ± 0.53	-	F(1,26)=7.537<br>**p=0.011**<br>**η²=0.225, d=1.078**
**Female**	0.93 ± 0.93	1.3 ± 0.73		F(1,26)=1.843<br>p=0.186<br>η²=0.066, d=0.532
Main effect:<br>*Time point*	-		Main interaction<br>*Gender*Time point*:<br>F(1,26)=8.79,<br>p=0.006<br>η²=0.253, d=0.271	
Simple effects:	F(1,26)=1.775, p=0.194<br>η²=0.064, d=0.523	F(1,26)=11.103,<br>**p=0.003**<br>**η²=0.299, d=1.306**		

Supplementary Table S2. Individual differences in sleep gain on ≥9AM-days. Linear regression analyses on sleep gain, sleep onset delay and sleep offset delay on ≥9AM-days compared to 8AM-days in cohort 2 (N=79). Abbreviations: b, unstandardized coefficient; std. error, standard error; beta, standardized coefficient; t, t-statistic; p, p-value. R^2 describes the explanatory power of the model (how much variance is explained). R^2 adjusted is the explanatory power accounted for the number of predictors in the model.

	**Sleep onset delay**					**Sleep offset delay**					**Sleep gain**				
**Predictors**	b	std. error	beta	t	p	b	std. error	beta	t	p	b	std. error	beta	t	p
(Intercept)	0.25	0.34	-0.30	0.72	0.471	1.27	0.31	0.24	4.10	<0.001	1.03	0.49	0.37	2.09	0.040
Gender: Male[a]	**0.53**	0.14	**0.91**	3.90	**<0.001**	0.01	0.12	0.02	0.07	0.942	**-0.52**	0.20	**-0.64**	-2.64	**0.010**
Grade level: 11[b]	-0.03	0.17	-0.05	-0.16	0.872	-0.21	0.16	-0.42	-1.33	0.186	-0.19	0.25	-0.23	-0.73	0.468
Grade level: 12[b]	0.01	0.18	0.03	0.08	0.933	-0.10	0.16	-0.20	-0.62	0.538	-0.11	0.26	-0.14	-0.45	0.655
Chronotype (MSF_{sc}; time in h)	-0.08	0.07	-0.12	-1.06	0.295	0.03	0.07	0.06	0.51	0.611	0.11	0.11	0.12	1.05	0.298
9AM-use (schooldays/ week)	0.04	0.06	0.06	0.55	0.583	-0.11	0.06	-0.21	-1.77	0.081	0.14	0.09	-0.17	-1.50	0.139
Observations	79					79					79				
R^2 / R^2 adjusted	0.175 / 0.119					0.072 / 0.008					0.113 / 0.052				

[a]Reference is female.
[b]Reference is grade level 10.

Supplementary Table S3. Sleep differences between time points and type of day in the longitudinal cohort (post-hoc comparisons relating to Fig. 6A). Two-way repeated measures ANOVAs were run for sleep onset, sleep offset, and sleep duration with the within-factors day (schooldays/weekends) and time point (t0/t1/t2) (see Fig. 6A). In case of significant interaction of both factors, simple effects (followed by Bonferroni-adjusted paired t-tests where indicated) are provided instead of the main effects. Data presented are mean ± standard deviation from the longitudinal cohort (n=33). η^2, (partial) eta squared; d, Cohen's d.

Sleep onset

	t0	t1	t2	Main effect: *Day*	Simple effects:	Paired t-tests:
**Schooldays**	-0.54h ± 0.79	-0.43h ± 0.75	-0.39h ± 0.73	-	F(2,31)=1.61<br>p=0.217<br>η^2=0.094, d=0.6442	-
**Weekends**	0.74h ± 0.94	0.46h ± 0.91	0.81h ± 1.00		F(2,31)=2.68<br>p=0.084<br>η^2=0.147, d=0.8303	-
Main effect: *Time point*	-			Main interaction *Day*Time point:*<br>F(2,64)=0.42<br>**p=0.020**<br>η^2=0.013, d=0.230		
Simple effects:	F(1,32)=153.70<br>p<0.001<br>η^2=0.823, d=4.313	F(1,32)=62.57<br>p<0.001<br>η^2=0.662, d=2.799	F(1,32)=72.88<br>p<0.00<br>η^2=0.695, d=3.019			

Sleep offset

	t0	t1	t2	Main effect: *Day*	Simple effects:	Paired t-tests:
**Schooldays**	6.62h ± 0.45	6.80h ± 0.47	6.76h ± 0.44	-	F(2,31)=9.03<br>**p=0.001**<br>**η^2=0.368,** d=1.526	t0-t1:<br>p<0.001<br>t0-t2:<br>p=0.025
**Weekends**	9.69h ± 0.97	9.31h ± 0.87	9.42h ± 0.93		F(2,31)=4.88<br>**p=0.014**<br>**η^2=0.240,** d=1.124	t0-t1:<br>p=0.004
Main effect: *Time point*	-			Main interaction *Day*Time point:*<br>F(2,64)=10.42<br>**p<0.001**<br>**η^2=0.246, d=1.142**		
Simple effects:	F(1,32)=294.21<br>**p<0.001**<br>**η^2=0.902, d=6.068**	F(1,32)=240.44<br>**p<0.001**<br>**η^2=0.883, d=5.494**	F(1,32)=322.85<br>**p<0.001**<br>**η^2=0.910, d=6.360**			

Sleep duration

	t0	t1	t2	Main effect: *Day*	Simple effects:	Paired t-tests:
**Schooldays**	7.10h ± 1.00	7.14h ± 0.56	7.16h ± 0.47	-	F(2,31)=0.54<br>p=0.588<br>η^2=0.034, d=0.375	**-**
**Weekends**	8.57h ± 0.42	8.50h ± 0.43	8.36h ± 0.55		F(2,31)=2.70<br>p=0.083<br>η^2=0.148, d=0.8336	**-**
Main effect: *Time point*	-			Main Interaction *Day*Time point*:<br>F(2,64)=3.88<br>**p=0.026**<br>**η^2=0.108, d=0.696**		
Simple effects:	F(1,32)=120.24<br>**p<0.001**<br>**η^2=0.790, d=3.879**	F(1,32)=96.52<br>**p<0.001**<br>**η^2=0.751, d=3.473**	F(1,32)=59.07<br>**p<0.001**<br>**η^2=0.649, d=2.720**			

Supplementary Table S4. **Sleep differences between schooldays and weekends in cohort 2.** Sleep onset, offset and duration from cohort 2 (n=105) at t2 are presented as mean ± standard deviation and were analysed via paired t-test. d_z, Cohen's d for paired t-tests.

	Sleep onset	Sleep offset	Sleep duration
**Schooldays**	-0.38h ± 0.81	6.83h ± 0.55	7.21 ± 0.76
**Weekends**	0.85h ± 1.11	9.57 ± 1.13	8.72h ± 0.95
**Paired t-test**	t(104)=-14.757, **p<0.001** **d_z=1.440**	t(104)=-26.471, **p<0.001** **d_z=2.583**	t(104)=-14.230, **p<0.001** **d_z=1.389**
